# Acknowledgement to Reviewers of *Marine Drugs* in 2017

**DOI:** 10.3390/md16010035

**Published:** 2018-01-17

**Authors:** Marine Drugs Editorial Office

**Affiliations:** MDPI AG, St. Alban-Anlage 66, 4052 Basel, Switzerland

Peer review is an essential part in the publication process, ensuring that *Marine Drugs* maintains high quality standards for its published papers. In 2017, a total of 389 papers were published in the journal. Thanks to the cooperation of our reviewers, the median time to first decision was 21 days and the median time to publication was 54 days. The editors would like to express their sincere gratitude to the following reviewers for their time and dedication in 2017:
Aarstad, Olav AndreasLeporatti, StefanoAbdel-Mohsen, A.M.Leung, Chung-HangAbraham, AnnLi, JieAccoroni, StefanoLi, NingAdunyah, Samuel E.Li, XiaohuaAeby, EricLiakos, Ioannis L.Afonso, Clélia N.Liaw, Chih-ChuangAgarwal, VinayakLin, Hai-shuAgostino, MarkLin, Wen-HanAgyei, DominicLin, Yen-ChangAiba, TetsuyaLin, ZhenjianAkanbi, TaiwoLindel, ThomasAlessandro, FanzaniLiou, Chian-JiunAlfonso, AmparoLiu, PinghuaAl-Horani, RamiLiva Rakotondraibe, HarinantenainaAlNajjar, MohammadLópez-Alonso, DiegoÁlvarez, MercedesLoughran, GaryAlves, CelsoLouzao, M. CarmenAlves, ElianaLowe, EdwardAmagata, TaroLu, JunAmillis, SotirisLu, JunAminin, Dmitry L.Lu, Mei-ChinAmsberg, Gunhild VonLu, Mei-ChinAndolfi, AnnaLu, YuananAndrianasolo, EricMacKenzie, LincolnAnraku, MakotoMacMillan, JohnApone, FabioMacrander, JasonAranaz, InmaculadaMaddau, LuciaAraoz, RómuloMadhukar, BurraArgia, KatsuhikoMaeda, HayatoArita, MinetaroMagarlamov, Timur Yu.Arizza, VincenzoMajumder, KaustavArmanini, DecioMaki, JamesAuer, AlexanderMalyarenko (Vishchuk), Olesya S.Avila-Roman, JavierMan, DulaAviles, Francesc XavierMancini, InesAzuma, KazuoMann, FrancisBácskay, IldikóMarco-Contelles, JoséBae, Hae-RahnMargassery, Lekha MenonBahorun, TheeshanMarino, AngelaBaker, BillMarrero, Ana R. DíazBakovic, MaricaMarti, ElisabetBakunina, IrinaMartin, JessicaBalasubramaniam, MeenakshisundaramMartín-Rodríguez, Alberto J.Ballottari, MatteoMaruyama, HirokoBanerjee, Hirendra NathMarverti, GaetanoBanerjee, MonimoyMarzocco, StefaniaBanskota, Arjun H.Matsuda, YudaiBarbosa, MarianaMatsugo, SeiichiBarchi, Joseph J.Matsumoto, TakuyaBarnathan, GillesMatsumura, ShuichiBarrajón-Catalán, EnriqueMatsuura, HiroshiBarratt, GillianMay, AaronBarrows, LouisMazur-Marzec, HannaBasti, LeilaMcculloch, MalcomBatista, IrineuMcDermid, Karla J.Bayer-Giraldi, MaddalenaMcHenry, PeterBazhenov, VasiliiMcMullin, David R.Bazire, AlexisMebs, DietrichBeberok, ArturMedina Torres, Miguel ÁngelBeckel, J. M.Mehbub, Mohammad FerdousBedini, EmilianoMehta, PoojaBellich, BarbaraMenzel, LorenzoBenediktsdóttir, Berglind EvaMerrick, MikeBergès, ThierryMeruvu, SunithaBernardi, FrancescoMessyasz, BeataBernards, MatthewMetcalf, JamesBeropoulis, EfthymiosMetsä-Ketelä, MikkoBerrue, FabriceMeyer, Kevin A.Bertin, SamuelMi, Fwu-LongBeukes, DenzilMichalak, IzabelaBianco, ArmandodorianoMikami, NanaBifulco, GiuseppeMiki, YasuhiroBirkemeyer, ClaudiaMiklossy, GabriellaBisignano, GiuseppeMilledge, John J.Bisson, JonathanMiller, John H.Bjørndal, BodilMimaki, YoshihiroBlanco, JuanMioso, RobertoBleve, GianlucaMiyamoto, YoheiBlokland, ArjanMiyashita, KazuoBlomme, EricMizuno, MasashiBlunt, JohnMoaddel, RuinBoddy, Christopher N.Mohanam, SanjeevaBohutskyi, PavloMolinaro, AntonioBoquet, DidierMonroig, OscarBorbone, NicolaMorabito, MarinaBorges, AnabelaMorabito, RossanaBorota, AnaMorales, Michael J.Bossier, PeterMorales-González, José AntonioBosso, LucianoMoreira, CristianaBotana, Ana M.Moreno, Antonio D.Botre, FrancescoMori, KiyoshiBotti, MaddalenaMori, NaokiBouaïcha, NoureddineMorikawa, ToshioBourdelais, AndreaMorimura, ShigeruBourguet-Kondracki, Marie-LiseMorioka, ShoBousbaa, HassanMorita, KyojiBoustie, JoëlMorita, NaokiBowden, BruceMoroney, James VBowman, MichaelMorris, GordonBownik, AdamMorse, DavidBraig, SimoneMorzycki, Jacek W.Brand, JacoMoschetta, MicheleBraquart-Varnier, ChristineMotti, CherieBreitenfeld Granadeiro, LuizaMuench, StephenBremer, ErhardMulder, Monique TBroggini, MassimoMüller, Werner E.G.Brown, Euan RMun, JiyoungBrown, LindsayMünch, JanBruzzone, SantinaMunday, RexBrzozowska, EwaMuramoto, KojiBučar, Dejan-KrešimirMurphy, Patrick J.Bucciarelli, Gary M.Murugaiyan, JayaseelanBurghardt, Kyle JonNakayama, YujiButler, MarkNam, Sang-JipCadamuro, MassimilianoNamba, TakushiCaffrey, PatrickNanjappa, DeepakCalado, RicardoNapier, JohnathanCampos Rosa, JoaquínNasopoulou, ConstantinaCanini, AntonellaNasri, RimCao, LingNeglia, GianlucaCapon, Robert J.Neilan, Brett A.Caramella, CarlaNeumann, TerrenceCaramujo, Maria JoséNewman, DavidCarballeira, Nèstor M.Ni, HongminCardona, FrancescaNicoletti, RosarioCardoso, JoãoNiksirat, HamidCarlisi, DanielaNishimura, ShinichiCarmeli, ShmuelNisticò, RobertoCarradori, SimoneNoguchi, TamaoCarrier, AliceNorouzitallab, ParisaCarvalho, PauloNorton, RayCasapullo, AgostinoNoshita, ToshiroCass, TonyNotbohm, HolgerCerasino, LeonardoNowaczyk, MarcCerella, ClaudiaNunomura, WataruCerra, BrunoO’Brien, KylieChan, Cheong XinO’Neil, GregoryChang, Ching-yaoOh, DongchanChang, Chun-JuOhta, ShinjiChang, Ko-TungOjika, MakotoChang, Long-SenOKADA, MasahiroChang, Shui-PingO'Keefe, Barry R.Chao, Louis KuopingOkino, TatsufumiChappell, KeithOliveira, Paula Alexandra Martins DeChen, GuoxunOnduka, ToshimitsuChen, HaixiaOpatz, TillChen, HongweiOrchard, SandraChen, Jiann-ChuOsman, NarinChen, LihuaOta, TsuguhiroChen, RongOtsuka, YuzuruChen, YiliangOzeki, YasuhiroChen, YingPagliara, PatriziaChen, ZhihangPalanisamy, Satheesh KumarCheng, Yuan-BinPan, Chun-HsuChevrot, RomainPandolfi, AssuntaChianese, GiuseppinaPanzella, LuciaChiesa, GiuliaPapadimitriou, Sofia A.Chifiruc, Mariana CarmenPapet, IsabelleChirumbolo, SalvatoreParikesit, Arli AdityaChiu, Tsai-HsinPark, WansuChoi, HyukjaeParsonage, DerekChoi, Jae SuePascale, AlbertoChoshi, TominariPatenaude, MathewChu, Ching-LiangPatruno, AntoniaChueh, Pin JuPattenden, GeraldChun, Kyung-SooPavic, ValentinaChung, Ying-ChienPedatella, SilvanaChung, Yong-AnPeigneur, SteveCicciu, MarcoPelin, MarcoCicero, ArrigoPence, BrandtCirrincione, GirolamoPennock, NathanCleary, Margot P.Penugonda, KavithaClericuzio, MarcoPereira, DavidCleveland, Beth M.Pereira, José AugustoCoates, Christopher JPereira, LeonelCobb, BrianPereira, LeonelColl, JosepPerepelyuk, MarynaCongestri, RobertaPérez, M.C.PinaCopolovici, Dana MariaPerez, Maria JoseCosta, FabíolaPerin, GiorgioCosta, Pedro ReisPerinelli, Diego RomanoCostantini, MariaPerrier, JosetteCostantino, ValeriaPescador, Sara CarbajoCrucho, Carina Isabel CorreiaPescitelli, GennaroCruz-Rivera, EdwinPestov, AlexandrCsaba, MáthéPetek, SylvainCustódio, LuísaPeter, MartinDa Gama, Bernardo A. P.Petitpas, Christian M.Dahiya, RajivPezzolesi, LauraDai, YuminPich, ChristineDaly, NorellePiggott, AndrewDaniels-Wells, Tracy R.Pillaiyar, ThanigaimalaiDaranas, Antonio HernándezPillay, VinessD'Argenio, ValeriaPinho, Brígida RibeiroDarkoh, CharlesPinto, Diana C. G. A.Darvin, Maxim E.Pires, CarlaD'Auria, ValeriaPitsikalis, MarinosDe Geest, BartPitsinos, EmmanuelDe La Cruz, Armah A.Poma, PaolaDe Mendonça, Dina I. M. D.Pombo, AnaDe Oliveira, Marcos RobertoPoniedziałek, BarbaraDe Pascale, DonatellaPoojary, MaheshaDe Petrocellis, LucianoPotterat, OlivierDe Tomaso, AnthonyPouchus, Yves-FrancoisDe Vendittis, EmmanuelePountos, IppokratisDefoirdt, TomPouponneau, PierreDella Sala, GerardoPozo, Óscar J.Den Hertog, JeroenPreece, Ellen P.Denaro, RenataProksch, PeterDennington, SimonProta, AndreaDenny, BillPucciarelli, SandraDeplazes, EvelynePuddick, JonathanDerby, CharlesPuglisi-Weening, MelanyDeshmukh, Sunil kumarPuiggalí, J.Devesa, VicentaPulido, DavidDiana, PatriziaPuri, AkshitDias Soeiro Cordeiro, Maria NatáliaPüschel, Gerhard P.Díaz, José FernandoPutt, KarsonDijkstra, Bauke W.Pytka, KarolinaDikalov, SergeyQaralleh, Haitham N.Diogène Fadini, JorgeQian, Pei-YuanDiretto, GianfrancoQueiroz, Maria-João R.P.Dishaw, LarryQuinn, Mark T.Domingos, Pedro M.Quinton, LoïcDominguez, HerminiaRace, PaulDonna Fu, ZidongRadwan, Mohamed M.Dormond, OlivierRahman, AzizurDorozhkin, SergeyRajauria, GauravDrago, Silvina RRalston, EmilyDrouiche, NadjibRämä, TeppoDuan, JingjingRamaraj, PanduranganDuggan, BrendanRamos-Vivas, JoséDuh, Chang-YihRana, DipakDwivedi, ChandradharRanasinghe, ShiwanthiDyshlovoy, SergeyRaposo, Maria Filomena De JesusDziewit, LukaszRateb, Mostafa E.Dziubla, ThomasRaymond, JamesEbrahim, HassanRebuffat, SylvieEbrahim, WeaamRecchi, ChiaraEhrlich, HermannRedecke, LarsEl Khalloufi, FatimaRein, KathleenEldarov, M. A.Reis, FlávioEldeeb, MohamedRepka, SariElShaer, AmrRescifina, AntonioElshahawi, SherifReveillon, DamienErdődi, FerencReyes, FernandoErmakova, SvetlanaReynolds, WilliamErnst, Robert K.Rho, Jung-RaeEsatbeyoglu, TubaRiesgo, AnaEscrig, Nuria CabedoRijo, PatríciaEsposito, Emilio XavierRinaudo, MargueriteEsteban, Maria AngelesRinge, DagmarEtxebeste, OierRittschof, DanielEvans, Bradley S.Rizzo, Angela MariaEvin, GenevièveRobb, CraigFaggio, CaterinaRobertson, AndrewFanizzi, Francesco P.Roca-Sanjuán, DanielFenical, WilliamRodrigues, FranciscaFernandes, CarlaRodriguez, JaimeFernandes, PedroRodríguez, JaimeFernandez Busquets, XavierRohde, KyleFernández, José J.Rokstad, Anne MariFesta, CarmenRomano, GiovannaFewer, DavidRosso, Marie-NoëlleFiedler, Hans-PeterRoué, MélanieFisher, Jed F.Rouhiainen, AriFitton, HelenRoullier, CatherineFleury, YannickRoussis, VassiliosFontana, AngeloRowley, Andrew F.Fouillaud, MireilleRubiolo, Pat6riziaFranco, ChrisRubis, BlazejFranco, ChrisRusso, PatriziaFranco, DiegoRzymski, PiotrFreitas, Ana C.Sabatier, Jean-MarcFrelek, JadwigaSaez, CarmenFrisvad, Jens C.Sahariah, PriyankaFrolova, LiliyaSaito, YoshiroFujii, RitsukoSakai, RyuichiFujimori, KoSakamoto, KensakuFujimoto, YukariSakamoto, ToshioFujita, MasakiSala, LucaFunato, HiromasaSalas, Jose A.G. Lago, João HenriqueSalim, VonnyGalasiti Kankanamalage, AnushkaSalmaso, NicoGanesan, A.Sansone, ClementinaGarces, EstherSarabia, FranciscoGarcia Vaquero, MarcoSassenhagen, IngridGarcía-Caballero, MelissaSatake, MasayukiGarcia-Camacho, FranciscoSatheesh, S.García-Mauriño, SofíaSato, ShigeruGasparri, FabioSatomi, YoshikoGatti, LauraSaurav, KumarGavagnin, MargheritaSawhney, NehaGavenonis, JasonSawyer, Thomas W.Gendaszewska-Darmach, EdytaScalise, MariafrancescaGenovese, GiuseppaSchaberle, Till F.Gentile, PiergiorgioSchaefer, MichaelGerdol, MarcoSchiraldi, ChiaraGerlach, JaredSchmalz, Hans-GuentherGerothanassis, Ioannis P.Schmid, J.Gilmour, JimSchmidt, AnkeGkelis, SpyrosSchols, DominiqueGnavi, GiorgioSchroeder, CristinaGobe, Glenda CSchroeder, GrzegorzGomes, Nelson G.M.Schupp, Peter J.Gonçalves, Lidia M DScognamiglio, MonicaGonçalves, M. Sameiro T.Scotti, LucianaGoncalves, OlivierSebastien, DutertreGonzález Maglio, Daniel H.Seca, Ana M. L.Gonzalez-Rey, ElenaSeifitokaldani, AliGörg, BorisSeipke, RyanGorman, ShelleySelan, LauraGórska, SabinaSeligmann, HervéGostner, Johanna M.Senter, Peter DGotor-Fernández, VicenteSeo, Jung-KilGraziano, GuellaSepčić, KristinaGrève, PierreSerra, StefanoGribble, Gordon W.Servent, DenisGrignani, GiovanniSeth, DevanshiGrkovic, Dr. TanjaShaaban, Khaled AGruber, ChristianShaikh, Mohd. FarooqGu, MeigangShen, Chia-NingGugerell, AlfredShen, Tang-LongGuitton, YannShenvi, RyanGulder, Tobias A. M.Sheu, Jyh-HorngGuriec, NathalieShikov, AlexanderGustafson, KirkShin, JongheonGutiérrez, AnaShinada, TetsuroGutiérrez-Juárez, RogerShiono, TakeshiGuzmán, Esther A.Shiozawa, YusukeHachiya, AkiraShishido, Tânia K.Hadwiger, LeeSiewert, BiankaHallegraeff, Gustaaf M.Silipo, AlbaHamaguchi, MasahideSilke, JoeHamann, MarkSim, RobertHammerl, Jens AndreSimionato, DianaHampel, DanielaSimon, Marie-ChristineHan, Quan-BinSimon-Yarza, TeresaHan, Sang-MiSimpson, TomHansen, EspenSingh, ArunimaHara, HiroshiSiodłak, DawidHarder, JensSivamani, Raja K.Harnedy, PadraiginSiwek, AgataHaron, MonaSkellam, ElizabethHarris, WilliamSleebs, Brad E.Harrison, Paul H. M.Slominski, AndrzejHartmann, AnjaSmid, Scott D.Harvey-Samuel, TimSmith, Judith A.Harwood, TimSmith, Thomas E.Hashimoto, WataruSolovchenko, AlexeiHatae, NoriyukiSolymosi, KatalinHattori, YuichiSon, Kwang-heeHaug, TorSonnenschein, EvaHayes, AlanSoule, TanyaHeras, AngelesSri Vedavyasa Sri, RadhakrishnanHerndon, DavidSrivastava, AnupHerrero, MiguelStacewicz-Sapuntzakis, MariaHlawaty, HannaStiger-Pouvreau, ValérieHoepfner, DominicStill, Patrick C.Hoffman, Angela M.Stojanovic, IvanaHojjat-Farsangi, MohammadStoskopf, MichaelHolland, Nolan B.Suenaga, KiyotakeHolst, OttoSugahara, TakuyaHolzel, ChristinaSugawara, AkihiroHonecker, FriedemannSuleria, Hafiz AnsarHong, PeiyingSumi, ShoichiroHong, ShuangsongSung, Ping-JyunHopkinson, BrianSupuran, Claudiu T.Hopping, GeneSuzuki, IwaneHorvath, David P.Sydnes, Magne O.Houen, GunnarSzcześ, AlexandraHsu, Fu-yinTacconelli, StefaniaHuang, Chi-ChangTaira, JunseiHuang, Chih-LingTajima, TakahisaHuerlimann, RogerTakahashi, DaisukeHungerford, JamesTakaichi, ShinichiHwang, Bang YeonTakayuki, TsukuiHwang, Pai-AnTanabe, YooIftime, DumitritaTanaka, NaonobuIguchi, TaisenTao, ShashaImperatore, ConcettaTartaglione, LucianaInuzuka, ToshiyasuTashima, ToshihikoIoanna, AndreadouTava, AldoIoannou, EfstathiaTavío, María Del MarIorga, B. I.Teicher, Beverly A.Isnansetyo, AlimTerent’ev, Alexander O.Ito, shosukeTeruya, KiichiroIto, TakuyaTester, PatriciaIttner, ArneTeta, RobertaIvankin, AndreyThakur, VijayIvanov, Alexander VThakur, Vijay KumarIwasaki, ArihiroThamilselvan, SivagnanamJakeman, David L.Theoduloz, CristinaJanczarek, MonicaThèvenod, FrankJanda, ElzbietaThomas, Eric J.Janouškovec, JanThomas, JimJanowski, MiroslawThomas, Olivier P.Jansen, RolfTidgewell, KevinJantas, D.Tietze, AlesiaJeng, Sen-ShyongTilney, Charles L.Jeon, OjuTitorenko, Vladimir I.Jeon, Taeck JoongTohru, KobayashiJeon, You JinToranzo, Alicia E.Ji, JamesTosti, ElisabettaJi, PengTownsend, Steven D.Jimbo, MitsuruTrincone, AntonioJiménez, CarlosTsai, Tsung-YuJirgensons, AigarsTsutsui, ShigeyukiJobling, MalcolmTurk, TomJohnsona, Rodney W.Turner, AndrewJoo, Hong-GuTutino, Maria LuisaJuliano, ClaudiaTyan, Yu-ChangJung, JeeUeda, KenjiJuszczak, LesławUeda, MitsuhiroKaas, QuentinUhrin, DusanKabala-Dzik, AgataUmeyama, AkemiKadam, ShekharUsuki, YoshinosukeKalinin, Vladimir I.Vaca, InmaculadaKallifatidis, GeorgiosVale, CarmenKamiya, MitsunobuVale, PauloKanakkanthara, ArunValentão, PatríciaKang, ChanKyuVan Der Meeren, AnneKang, HeeVan Lanen, StevenKang, Heon-joongVarga, ElisabethKanno, YuichiroVasconcelos, VítorKashino, YasuhiroVassilevski, AlexanderKashman, YoelVázquez, José AntonioKasten-Jolly, JaneVentura, TomerKatikou, PanagiotaVepsäläinen, JoukoKato, Takamitsu A.Verestiuc, LilianaKatsifas, Efstathios A.Viseras, CesarKawai, ShigeyukiVon Salm, JacquelineKawamura, AkiraVon Schacky, ClemensKem, William R.Voruganti, SukeshKenney, JaniceWahlsten, MattiKerch, GarryWalsh, DavidKerchev, PavelWalsh, William R.Kerr, RusselWang, Hui-ChunKeyzers, RobWang, PengKhachik, FrederickWang, PiwenKhalil, ZeinabWang, San-LangKhan, MohsinWang, San-LangKhozin-Goldberg, InnaWang, XiaohongKikuchi, HaruhisaWase, NishikantKikuchi, TakashiWatts, JoyKim, Chung SubWęgrzyn, GrzegorzKim, Ji YeonWeiergräber, MarcoKim, Jin-KyungWen, XinKim, Kyung-suWendling, Carolin C.Kim, LouWest, LyndonKim, Yong SooWhalen, KristenKishimoto, YoshimiWhite, Robert L.Kisselev, AlexeiWhyte, ClaireKlarskov, KlausWickenden, Alan D.Kleinteich, JuliaWickliffe, JeffreyKlettner, AlexaWilliams, DanielKlontzas, MichaelWilliams, KatherineKluza, JéromeWilliams, Leonard L.Knölker, Hans-JoachimWink, JoachimKobayashi, ToyoharuWinterhalter, MathiasKobayashi, YoshiroWłodarczyk, MaciejKogure, KentaroWozniak-Knopp, GordanaKöhn, MajaWu, Chang-JerKoller, MartinWu, Chang-YiKomaki, HisayukiWu, Chi-ReiKondo, HidemasaWu, Li-ChenKorkina, Ludmila G.Wu, Tzu-HuaKoshino, HiroyukiWu, Yang-ChangKoskela, Harri T.Xi, LinKotoku, NaoyukiXing, FeiKourist, RobertXu, MiaoKourkoumelis, NikolaosYagi, HisashiKoziel, JacekYamada, SohsukeKretzschmar, Anna LizaYamaki, KohjiKruger, JacobYamashita, EijiKrupa-Kozak, UrszulaYamazaki, TomomiKuan, Yu-HsiangYan, NingKubota, TakaakiYang, JiaoKuksis, ArnisYang, QiyuanKunte, Hans-JörgYang, Wei-hsiungKuo, Ping-ChungYaoita, YasunoriKurihara, HideyukiYasukawa, KiyoshiKwan, Hiu-yeeYi, MyunggiKwan, JasonYokosuka, AkihitoKweon, Oh-KyeongYokoya, MasashiLa Regina, GiuseppeYoon, Do-YoungLacaille-Dubois, Marie-alethYotsu-Yamashita, MariLambrinidis, GeorgeYu, RongminLang, SiegmundYurchenko, AntonLászló, KöhidaiZabetakis, IoannisLauritano, ChiaraZahonova, KristinaLava, Sebastiano A.G.Zanolla, MarianelaLavrik, OlgaZeilinger, CarstenLaycock, BronwynZhang, DeqiangLazado, Carlo C.Zhang, FanLee, ChangWooZhang, FumingLee, Ching-KuoZhang, JunzengLee, Jae KwonZhang, KaiLee, Jong SukZhang, QinghaiLee, Kyun-WooZhu, XuejunLee, Shiow-JuZhukova, NataliaLee, VictorZovko, AnaLegros, ChristianZubia, EvaLemmens-Gruber, RosaZuccaro, AlgaLeone, AntonellaZuo, LiLeonidas, Demetres D.Zutz, ChristophLeón-Rivera, IsmaelZuvela, PetarLepiarczyk, Ewa


